# Evaluation of OvaCyte for the detection of gastrointestinal parasites in ovine and bovine animals: comparison with traditional flotation techniques

**DOI:** 10.1051/parasite/2026002

**Published:** 2026-02-24

**Authors:** Nagwa Elghryani, Geetika Lahan, Jayanta Bor Gohain, Eileen Collins, Trish McOwan, Theo de Waal

**Affiliations:** 1 Telenostic Limited R95 WN20 Kilkenny Ireland; 2 Department of Biology, Faculty of Arts and Sciences-Gamines, University of Benghazi 33FX + QV9 Benghazi Libya; 3 School of Veterinary Medicine, University College Dublin D04 D6F6 Dublin Ireland

**Keywords:** Faecal egg count, *Haemonchus contortus*, Trichostrongylidae, *Nematodirus battus*, Precision, Ruminant livestock

## Abstract

This study aimed to evaluate the diagnostic and analytical performance of OvaCyte, an automated image-based system for the detection of gastrointestinal parasites in ruminants, against traditional flotation techniques. OvaCyte is available in two versions: OvaCyte Plus, which automates egg detection and quantification, and OvaCyte Premium, which incorporates enhanced analysis to differentiate parasite families, genera, and species within strongyles (*e.g.*, Trichostrongylidae, *Haemonchus contortus*, and *Nematodirus* spp.). Coccidia are also classified as standard or type B, with the latter specifically including *Eimeria weybridgensis, Eimeria crandallis* and *Eimeria ovinoidalis*, based on distinct morphometrical features. The identification of *Haemonchus contortus* was validated using peanut agglutinin (PNA) fluorescence staining as the gold standard. Sensitivity and specificity for OvaCyte Plus, Mini-FLOTAC, and McMaster were calculated based on a consensus “true status”. Limits of detection and quantification were calculated using regression analysis. OvaCyte Plus demonstrated the highest sensitivity, especially for *Nematodirus* (95%), coccidia (93%), strongyles (92%), and *Strongyloides papillosus* (90%). Mini-FLOTAC showed moderate sensitivity (63–79%), while McMaster had the lowest value (30–76%). For *Moniezia* spp., sensitivity was similar for OvaCyte Plus and Mini-FLOTAC (79%), but lower for McMaster (59%). Specificity was high across all techniques (92–100%). Differences in performance were attributed to varying multiplication factors: OvaCyte Plus (3 EPG), Mini-FLOTAC (7.5 EPG), and McMaster (50 EPG). Strong correlations were observed between OvaCyte Plus and manual techniques for strongyle egg counts. OvaCyte Premium exhibited the highest sensitivity across all parasites. OvaCyte Plus and Premium demonstrated performance comparable to or exceeding traditional techniques for the detection of gastrointestinal parasites in ruminants.

## Abstract (fr)

Cette étude visait à évaluer les performances diagnostiques et analytiques d’OvaCyte, un système automatisé d’imagerie pour la détection des parasites gastro-intestinaux chez les ruminants, par rapport aux techniques de flottaison traditionnelles. OvaCyte est disponible en deux versions : OvaCyte Plus, qui automatise la détection et la quantification des œufs, et OvaCyte Premium, qui intègre une analyse avancée pour la différenciation des espèces et des familles de parasites, notamment parmi les strongles (par exemple Trichostrongylidae, *Haemonchus contortus* et *Nematodirus* spp.). Les coccidies sont également classées en deux types : standard et de type B, ce dernier incluant spécifiquement *Eimeria weybridgensis*, *Eimeria crandallis* et *Eimeria ovinoidalis*, selon des caractéristiques morphométriques distinctes. L’identification d’*Haemonchus contortus* a été validée par coloration fluorescente à l’agglutinine d’arachide (PNA), considérée comme la méthode de référence. La sensibilité et la spécificité des tests OvaCyte Plus, Mini-FLOTAC et McMaster ont été calculées selon un consensus sur le statut réel des parasites. Les limites de détection et de quantification ont été calculées par analyse de régression. OvaCyte Plus a démontré la plus grande sensibilité, notamment pour *Nematodirus* (95 %), les coccidies (93 %), les strongles (92 %) et *Strongyloides papillosus* (90 %). Mini-FLOTAC a montré une sensibilité modérée (63–79 %), tandis que McMaster a présenté la plus faible (30–76 %). Pour *Moniezia* spp., la sensibilité était similaire pour OvaCyte Plus et Mini-FLOTAC (79 %), mais inférieure pour McMaster (59 %). La spécificité était élevée pour toutes les techniques (92–100 %). Les différences de performance ont été attribuées aux différents facteurs de multiplication : OvaCyte Plus (3 œufs par gramme), Mini-FLOTAC (7,5 œufs par gramme) et McMaster (50 œufs par gramme). De fortes corrélations ont été observées entre OvaCyte Plus et les techniques manuelles pour le comptage des œufs de strongles. OvaCyte Premium a présenté la plus grande sensibilité pour tous les parasites. OvaCyte Plus et Premium ont démontré des performances comparables, voire supérieures, aux techniques traditionnelles de détection des parasites gastro-intestinaux chez les ruminants.

## Introduction

Parasitic diseases pose a significant health constraint on the efficient production of livestock, with gastrointestinal (GI) helminth and protozoan infections having a notable welfare impact globally [10]. Clinical signs of infection typically include malnutrition, weight loss, diarrhoea, anaemia, and even death, and considerable production losses. This is further exacerbated by the increasing rate of drug resistance, particularly in nematodes [26, 29]. The economic burden of these diseases is estimated to be in the tens of billions of dollars annually [9].

GI nematodes and coccidia infections in grazing ruminants represent a diverse range of parasitic genera and species, with nematode species such as *Haemonchus contortus*, *Ostertagia ostertagi*, *Cooperia oncophora*, *Teladorsagia circumcincta*, and *Trichostrongylus* spp. being of the highest economic importance in Ireland and Europe [9, 42]. These Trichostrongylidae significantly contribute to the challenges faced in livestock production. Shifting disease patterns and challenges in controlling parasitic infections highlight the need for risk-based approaches to manage major parasitic diseases in sheep and cattle [5, 42]. Early and accurate diagnosis is crucial for guiding treatments, delaying anthelmintic resistance, and mitigating the impact of infections [17, 39]. The increasing challenge of anthelmintic resistance threatens effective parasite control, reducing the success of treatments [33]. Traditional faecal egg count (FEC) techniques, although effective for estimating GI nematode infection levels, often fail to accurately distinguish between morphologically overlapping species and therefore rely on larval culture techniques for accurate species-level identification. These techniques are often time-consuming, and lack sensitivity, particularly in cases of low-intensity infections [28]. However, new diagnostic techniques such as molecular techniques and next generation sequencing should be developed that can enhance the precision of species identification, enabling more targeted and effective treatment strategies, each targeting a species of interest [28, 31, 32]. The proportions of infective species detected can then be assessed and used to determine the most effective treatment. Recently, Ljungström *et al.* [22] used the McMaster (MCM) technique to quantify the number of *H. contortus* eggs in the sample with success, and while both peanut agglutinin (PNA) staining and molecular techniques were more sensitive than microscopy, results show that microscopy can offer a viable alternative to these techniques, especially when only estimation is required. Molecular techniques using polymerase chain reaction (PCR) or next generation sequencing are also available for species identification and quantification [22, 43]. Fluorescently labelled PNA has previously been shown to specifically bind to the eggs of *H. contortus*, enabling their identification using fluorescence microscopy [27]. However, these techniques are labour-intensive and require expensive, specialised equipment for analysis, which are not accessible to local veterinary practitioners.

These challenges highlight the importance of on-site real-time analysis, as a faster and more accessible alternative for farmers and veterinary practitioners. Recent advancements in artificial intelligence (AI) and deep learning have made it feasible to identify parasitic eggs and oocysts based on their morphological characteristics. Over the past few years, OvaCyte Telenostic (OCT) has actively developed AI-driven models capable of detecting the most prevalent GI parasites in bovine and equine hosts, utilising image-based recognition of key morphological features [14, 15]. Furthermore, the OvaCyte Pet system represents a recent innovation developed for the identification of GI parasites in companion animals [16].

At the genus level, the identification of parasitic elements is achievable for AI algorithms, as many common parasitic elements possess distinctive morphological traits that facilitate classification. However, greater complexity arises when attempting species-level identification due to morphological similarities among certain taxa. To address this, Telenostic has developed two new and advanced automated systems: OvaCyte Telenostic Plus (OCT^+^), and OCT Premium. These are AI-powered advanced versions of the OvaCyte Telenostic device [14, 15]. The OCT^+^ diagnostic system is designed to analyse the most significant GI parasites in cattle and sheep, including strongyles, coccidia, *Nematodirus* spp., *Moniezia* spp., and *Strongyloides* spp. This advancement has been achieved by modification to the cassette, with increased volume analysed in the channel, from 2 mL to 7 mL of filtrate. OCT Premium offers an addition computational evaluation for ovine faecal scan images to differentiate the species/family, as described by Taylor *et al.* [38], within the strongyle group [Trichostrongylidae, *H. contortus*, *Nematodirus* (*Nematodirus battus* and *Nematodirus* spp*.*)], and coccidia, broadly grouped into “coccidia” and “coccidia type B”, respectively. “Coccidia type B” detects only *Eimeria weybridgensis*, *Eimeria crandallis* and *Eimeria ovinoidalis*. OCT Premium is designed for rapid morphometrical analysis of GI parasitic eggs/oocyst. This includes analytical features such as symmetry, major and minor axes, and aspect ratio. Both systems systematically scan a cassette filled with an animal faecal solution, capturing high-resolution images to identify and quantify the presence of parasitic elements, providing accurate FECs expressed as eggs/oocysts per gram (EPG/OPG). Utilising deep learning-based algorithms, it delivers quantitative results in a short time frame compared to microscopic examination of faecal samples, which can be subject to variability depending on the operator’s level of training and experience [4, 28]. Therefore, the purpose of this study was to validate the performance of OCT^+^ and OCT Premium on both a diagnostic and analytical level, in comparison to the conventional microscopic parasitology techniques used in veterinary laboratories. Diagnostic performance was assessed based on the sensitivity (Se) and specificity (Sp) of each technique in detecting individual parasitic species. Analytical performance was evaluated by examining the precision of the results generated by each technique, and determining whether they exhibited consistent results across different levels of parasite concentrations, thereby confirming their quantitative reliability. The study objectives are outlined as follows:

- To evaluate the diagnostic and analytical performance of the OCT^+^ system for the quantitative and qualitative detection of specific GI parasites in bovine and ovine faecal samples, in comparison with validated traditional microscopic diagnostic techniques.
- To evaluate the performance of the automated predictions of the OCT Premium product for further species identification of Trichostrongylidae, *Nematodirus*, and coccidia within ovine faecal specimens against both existing conventional parasitological techniques and by PNA fluorescence staining for *H. contortus* only.

## Materials and methods

### Introduction to techniques

A summary of the techniques used in this study is presented in Table 1.

**Table 1:** Brief introduction to the techniques used in this study.

Technique	Abbreviation	Host faecal sample (number of samples)	Site of execution	Volume of sample (g)	Detection limit (EPG/OPG)	Parasite identified
OvaCyte Plus	OCT^+^	Sheep (103) and cattle (86)	UCD	3	3	Strongyles, coccidia, *Nematodirus* spp., *Moniezia* spp., and *Strongyloides papillosus*
Premium	OCT Premium	Sheep (40)	UCD	3	3	Strongyle group (*Trichostrongylus*, *Haemonchus contortus*, *Ostertagia, Teladorsagia*, *Chabertia*, *Oesophagostomum*, *Cooperia*, *Nematodirus* (*Nematodirus* spp. and *Nematodirus battus*)), and coccidia (coccidia and coccidia type B (*Eimeria weybridgensis*, *Eimeria crandallis*, and *Eimeria ovinoidalis*)).
McMaster	MCM	Sheep (103) and cattle (86)	UCD	3	50	Strongyles, coccidia, *Nematodirus* spp., *Moniezia* spp., and *Strongyloides papillosus*
Mini-FLOTAC	MF	Sheep (103) and cattle (86)	UCD	3	7.5	Strongyles, coccidia, *Nematodirus* spp., *Moniezia* spp., and *Strongyloides papillosus*
McMaster at Laboratory A	MCM Lab A	Sheep (40)	Sweden	–	50	*Haemonchus contortus*, Trichostrongylidae, *Nematodirus battus*, and coccidia type B (*Eimeria weybridgensis*, *Eimeria crandallis*, and *Eimeria ovinoidalis*)
McMaster at Laboratory B	MCM Lab B	Sheep (40)	Sweden	–	50	Trichostrongylidae, *Nematodirus battus*, and coccidia type B (*Eimeria weybridgensis*, *Eimeria crandallis*, and *Eimeria ovinoidalis*)
Peanut Agglutinin lectin staining	PNA	Sheep (40)	UCD	4	–	*Haemonchus contortus*

UCD = University College Dublin.

### Sample collection

Fresh cattle and sheep faecal samples were collected from the ground on farms in Ireland, between December 2024 and February 2025. Only the superficial layer was collected to minimise environmental contamination. The samples were then potted, labelled, and preserved in sodium acetate-acetic acid-formalin (SAF) solution at the Telenostic laboratory before being transferred to the Parasitology laboratory at University College Dublin (UCD) for analysis.

A total of 189 faecal samples (~50 g each) were collected from sheep (*n* = 103) and cattle (*n* = 86) to evaluate the diagnostic performance of OCT^***+***^, MCM, and Mini-FLOTAC (MF). For analytical performance, a composite sheep faecal sample was prepared by pooling approximately 5 g from each of six ovine samples with high egg-EPG levels.

The diagnostic performance of OCT Premium was evaluated using 40 sheep faecal samples. Each sample was divided into four aliquots for parallel analysis: two were analysed at UCD for OCT Premium and the PNA techniques, while the remaining two were sent to two independent accredited laboratories in Sweden: laboratory A (Lab A) and laboratory B (Lab B). Additionally, to assess the analytical performance of OCT Premium in comparison to MCM Lab A, precision testing was performed on a sheep faecal sample. This sample was divided into two aliquots of at least 50 g each. One aliquot was sent for analysis to Lab A in Sweden, and the other aliquot was analysed approximately three days later with the OCT Premium at UCD, Figure 1.

**Figure 1:**
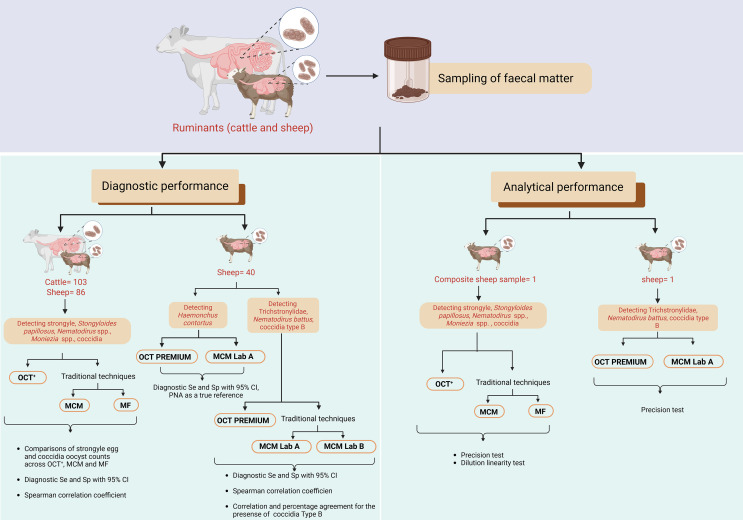
Schematic representation of the study, illustrating the workflow for diagnostic and analytical performance, where OCT^+^ refers to OvaCyte Plus, OCT Premium to OvaCyte Premium, MCM to McMaster technique, MF to Mini-FLOTAC, MCM Lab A to McMaster performed at laboratory A, MCM Lab B to McMaster performed at laboratory B, and PNA to peanut agglutinin fluorescence staining. Se indicates sensitivity and Sp indicates specificity, both with 95% confidence intervals (CI).

### Diagnostic performance

#### Sample preparation and test procedure

Each sample was well homogenised with the help of a spatula before testing for the uniform distribution of the parasite eggs. For diagnostic performance of OCT^+^, MCM, and MF, approximately 3 g of faecal matter were mixed thoroughly with 42 mL of saturated sodium chloride solution [NaCl, specific gravity (S.G.) 1.2]. The suspension was then filtered through an OvaCyte wire mesh filter. The same filtrate was then used for each of the three FEC techniques [15]. FECs were carried out using an MCM slide with a detection limit of 50 EPG [23], an MF slide (detection limit: 7.5 EPG) [12], and the OCT^+^ technique (detection limit: 3 EPG). The cassette used for OCT^+^ was the new higher volume 7 mL version. For the diagnostic performance of OCT Premium, the filtrate was prepared using the same procedure as OCT^+^, while MCM Lab A and MCM Lab B followed their respective standard operating protocol. The PNA technique as described by Jurasek *et al.* [19] was performed on the 40 ovine faecal samples using PNA lectin stain (Sigma, Cat. No. L-7381; derived from *Arachis hypogaea* diluted in deionised water at 1 mg/1 mL) and samples showing fluorescent strongyle eggs were identified under the fluorescence microscope (Nikon Eclipse E400 with CoolLED pE-300 Controller and FITC filter [excitation: 465–495 nm, dichroic Mirror: 505 nm, Bandpass: 515–555 nm; Nikon Instruments, Tokyo, Japan; CoolLED Ltd., Andover, UK)]. These samples were considered positive for the presence of *H. contortus*. Images of fluorescent strongyle eggs in each positive sample were taken for reference (see supplementary data).

### Analytical performance

#### Precision test

The precision test for OCT^+^, MCM, and MF was carried out using one composite sheep sample weighing approximately 35 g, tested in 10 replicates. Each of the 10 sample repetitions involved a freshly prepared sample which was then processed using all three techniques to ensure consistency and an accurate assessment of precision. Similarly, to compare the precision of OCT Premium and MCM Lab A, a thoroughly homogenised sample was analysed in 10 replicates at respective locations. The sample preparation for each technique followed the protocol outlined above (“Sample preparation and test procedure”).

#### Dilutional linearity test

The composite sheep sample used in the precision test was further analysed using the three diagnostic techniques: MCM, MF, and OCT^**+**^. Samples (6 g of faecal matter diluted in 84 mL of flotation fluid; NaCl S.G. 1.2) were prepared following the methodology described above (“Sample preparation and test procedure”), with each analysis performed in triplicate. After filling the OCT^+^ cassette, MCM slide, and MF disc, the remaining filtrate of the sample was diluted with flotation fluid in a 1:9 ratio (10 mL of the sample filtrate in 90 mL of flotation fluid) and analysed again in triplicate. Serial 1:9 dilutions of the original sample were continued and analysed in triplicate until no parasite eggs were detected for any species.

### Statistical analysis

Comparisons of strongyle egg and coccidia oocyst counts across OCT^+^, MCM, and MF were conducted using R (version 4.4.2) [30], by evaluating median count differences within stratified MF technique-based count bands. The diagnostic Se and Sp of the OCT^+^, MCM, and MF were calculated against the ‘true status’ of each sample for each parasitic species, using Microsoft Excel (Version 2502). The true status of a sample was considered ‘positive’ if any of the three technique tested positive, and ‘negative’ if all tested negative for each parasite species. Similarly, Se and Sp of the OCT Premium, MCM Lab A, and MCM Lab B at two threshold levels: at 50 EPG cut-off (OCTPre50) and at 3 EPG cut-off (OCTPre3), were calculated for the detection of *N. battus*, Trichostrongylidae, and coccidia type B, employing the same criteria for determining true status. For the detection of *H. contortus* at the two thresholds, OCT Premium was compared only against MCM Lab A, while the PNA technique served as the true reference. The 95% confidence intervals were calculated using the Clopper-Pearson method for binomial proportions. Two EPG thresholds were applied for OCT: 50 EPG, to match the threshold of the MCM technique, and 3 EPG, to assess performance at lower infection levels.

To assess the agreement between OCT^+^, MCM, and MF, for strongyle detection, Spearman correlation coefficients were calculated in SPSS 29 (IBM SPSS Statistics). The results were considered statistically significant at *p* < 0.05. The quantitative correlation between Trichostrongylidae (at OCTPre50 and OCTPre3) and coccidia OPG was similarly evaluated between OCT Premium, MCM Lab A, and MCM Lab B. For binary detection of coccidia type B, agreement between methods was assessed using pairwise agreement rates, expressed as both absolute agreement numbers and as percentages. To further assess diagnostic concordance, 2 × 2 confusion matrices and Cohen’s Kappa coefficients were computed using R. Agreement between each pair of diagnostic methods (OCT^+^, MCM, and MF) reflects how consistently the techniques classified samples as either positive or negative for each parasite.

The analytical performance of OCT^+^, MCM, and MF was evaluated from precision and dilution linearity tests, while that of OCT Premium and MCM Lab A was evaluated on precision test only. Linear regression was also performed in Excel to provide complementary information, such as the R-squared value, and to calculate the limit of detection (LOD) and limit of quantification (LOQ). The LOD and LOQ were calculated using the standard formulas (3.3*(σ/s)) and (10*(σ/s)), respectively, where σ is the standard deviation (SD) of the intercept and ‘s’ is the slope of the regression line.

## Results

### Strongyles and coccidia egg count comparisons using the three different techniques: OCT^+^, MCM, and MF

The median count differences in coccidia oocysts and strongyle egg counts between each of the three techniques according to the number of oocyst/egg bands, measured by MF, are presented in Figure 2. These differences are shown across defined MF oocyst/egg count ranges to illustrate how each method performs relative to MF at varying levels of parasite counts. At low count levels (0–100), both MCM and OCT^+^ produced values comparable to MF. As parasite counts increased, however, the degree of divergence between methods became more evident. MCM consistently underestimated relative to MF, with the highest degree of underestimation observed in the 501–1000 and 1001–2000 ranges. In contrast, OCT^+^ yielded higher counts than MF at moderate and high count levels.

**Figure 2:**
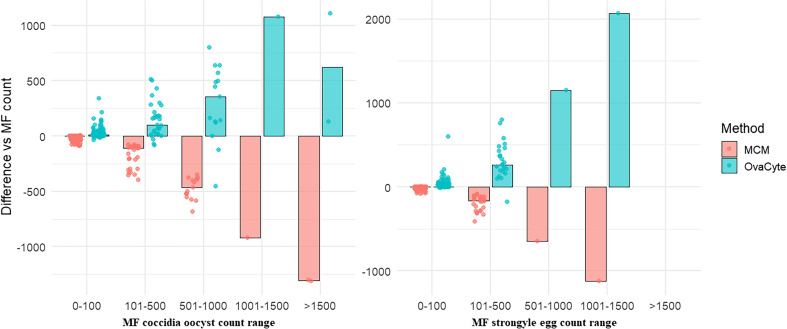
Comparative bar plots illustrating the median count differences for coccidia oocyst (A) and strongyle egg (B) counts between OvaCyte Plus (OCT^+^) and McMaster (MCM), compared to Mini-FLOTAC (MF) at different MF egg/oocyst count ranges.

### Diagnostic performance

#### Diagnostic Se and Sp of the OCT^+^ product in comparison to MCM and MF for detecting gastrointestinal parasites

The Se and Sp of three different techniques for detecting various parasites are shown in Table 2. The OCT^+^ technique demonstrated the highest Se across all parasites (79% to 95%) with the highest values for *Nematodirus* spp. (95%), coccidia (94%), strongyles (92%), and *S. papillosus* (90%). MF showed moderate Se (63–79%), while MCM had the lowest (30–76%). For *Moniezia* spp., Se was similar for OCT^+^ and MF (79%), but lower for MCM (59%), while Sp remained high across all methods (95–100%).

**Table 2:** Sensitivity (Se) and specificity (Sp) with 95% confidence intervals (CI%) of the three techniques (OCT^+^: OvaCyte Plus, MCM: McMaster, and MF: Mini-FLOTAC) for gastrointestinal parasites in ruminant (sheep and cattle) faecal samples.

Parasite	Se and Sp	Technique		
		OCT ^+^	MCM	MF
Coccidia	Se	94% (88–97)	76% (68–83)	87% (80–92)
	Sp	100% (93–100)	100% (93–100)	100% (93–100)
Strongyles	Se	92% (87–96)	63% (55–71)	86% (79–91)
	Sp	94% (82–99)	100% (92–100)	100% (92–100)
*Nematodirus* spp*.*	Se	95% (84–99)	52% (36–68)	79% (63–90)
	Sp	94% (97–100)	100% (99.9–100)	100% (99.9–100)
*Moniezia* spp*.*	Se	79% (66–88)	59% (45–72)	79% (66–88)
	Sp	95% (90–98)	100% (97–100)	100% (97–100)
*Strongyloides papillosus*	Se	90% (82–95)	30% (21–40)	63% (53–72)
	Sp	92% (84–97)	100% (96–100)	100% (96–100)

#### Diagnostic sensitivity of OCT Premium (OCTPre50 and OCTPre3), MCM Lab A, and MCM Lab B for the detection of *N. battus* and Trichostrongylidae

The diagnostic Se of OCT Premium (OCTPre50 and OCTPre3), MCM Lab A, and MCM Lab B for the detection of *N. battus* and Trichostrongylidae is presented in Table 3. Of the samples analysed, 11 were positive and 29 were negative for *N. battus*. At a cut-off 3 EPG, OCT premium had an Se of 91% and Sp of 100%. However, when the cut-off was increased to 50 EPG, the Se of OCT Premium dropped to 0%, with the Sp remaining at 100%. Both MCM Lab A and MCM Lab B achieved an Se of 18%, indicating limited diagnostic performance compared to OCT Premium. For Trichostrongylidae, no true negative samples were identified; therefore, Sp could not be calculated. OCT Premium consistently demonstrated high Se for the detection of Trichostrongylidae across both cut-off points. At a cut-off above 50 EPG, OCT Premium had an Se of 100%, while MCM Lab B and MCM Lab A showed a slightly lower Se of 93% and 89%, respectively. OCT Premium maintained an Se of 100% even at lower threshold (3 EPG), indicating its high diagnostic performance even at low egg counts. Some scanned images from OCT Premium, identifying *Trichostrongylus* spp. and *N. battus*, *H. contortus*, and coccidia and coccidia type B are shown in Figure 3.

**Figure 3:**
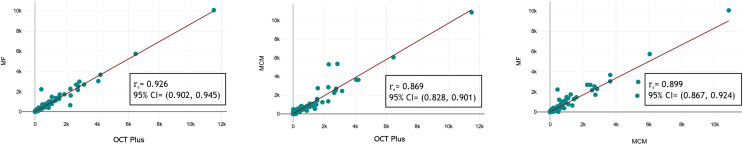
Scanned image of a *Trichostrongylus* spp. and *Nematodirus battus* (a), *Haemonchus contortus* (b), and coccidia and coccidia type B (c) egg from OCT Premium, with the bounding box.

**Table 3:** Sensitivity (Se) and specificity (Sp) with 95% confidence intervals (CI%) of the techniques (OCTPre3: OCT Premium at 3 EPG cut-off, OCTPre50: OCT Premium at 50 EPG cut-off, MCM Lab A: McMaster performed at laboratory A, and MCM Lab B: McMaster performed at laboratory B, and PNA: peanut agglutinin lectin staining) for the detection of *Nematodirus battus*, *Haemonchus contortus*, and Trichostrongylidae, where *n*^+^ is the number of true positive and n^-^ is the number of true negative samples.

Parasite	Se and Sp	Technique				
		OCTPre3	OCTPre50	MCM Lab A	MCM Lab B	PNA
*N. battus*						
*n*^+^ = 11	Se	91% (59–100)	00% (0–29)	18% (02–52)	18% (02–52)	NA
*n*^−^ = 29	Sp	100% (88–100)	100% (88–100)	100% (88–100)	100% (88–100)	NA
*H. contortus*						
*n*^+^ = 31	Se	93.5% (79–99)	54.8% (36–73)	06.5% (00–21)	N/A	100% (89–100
*n*^−^ = 9	Sp	77.8% (40–97)	100% (66–100)	100% (66–100)	N/A	100% (66–100)
Trichostrongylidae						
*n*^+^ = 40	Se	100% (91–100)	100% (88–100)	89% (72–98)	93% (76–99)	NA
*n*^−^ = 0						

#### Diagnostic sensitivity of OCT Premium (at OCTPre50 and OCTPre3), and MCM Lab A for the detection of *H. contortus*.

The diagnostic Se and Sp of OCT Premium (OCTPre50 and OCTPre3) and MCM Lab A for the detection of *H. contortus* is presented in Table 3. Based on the PNA technique used to determine the true status of *H. contortus* positive samples, 9 were confirmed to be negative and 31 positive. Examples of PNA fluorescent eggs from some of the positive samples are shown in Figure 4. The rest of the images are included in the supplementary data.

**Figure 4:**
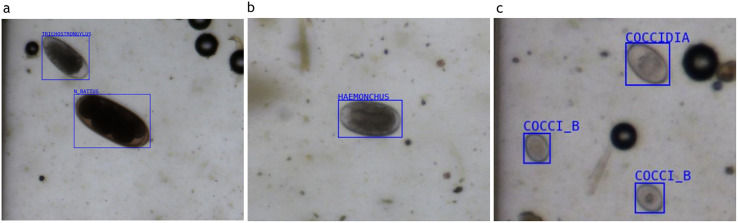
Microscopy images of *Haemonchus contortus* (indicated by the red arrow) with other strongyle eggs (indicated by the white arrow). Images were taken with (left) and without (right) UV light at (a) 200× magnification and at (b) 100× magnification, using fluorescent microscope (Nikon pE-300 Controller with FITC filter, manufactured in the UK).

The OCTPre3 demonstrated an Se and Sp of 93% and 100%, respectively. In contrast, when the OCTPre50 (theoretical cut-off applied) was applied, the Se decreased to 53%, while the Sp remained at 100%. The Se of MCM lab A was 7%, with an Sp of 100%n as presented in Table 3.

### Correlation

#### Correlation between OCT^+^, MCM, and MF for strongyle egg counts

**Figure 5:**
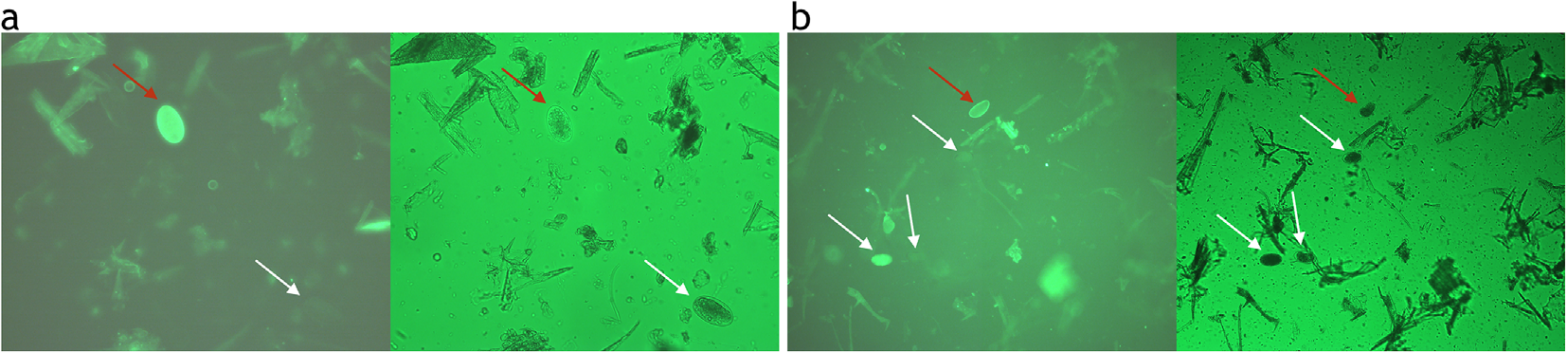
Spearman correlation analysis for strongyle egg counts EPG (eggs per gram) between OCT Plus, McMaster (MCM) and Mini-FLOTAC (MF). The Spearman correlation coefficient (*r*_*s*_) with 95% confidence interval is shown for each pair.

The correlation (*r*_*s*_) with 95% CI for strongyle eggs counts between OCT^+^, MCM, and MF is present in Figure 5. The comparison of OCT^+^ with other techniques for strongyle egg counts revealed a stronger correlation with MF (0.926) than with MCM (0.869), while the correlation between MF and MCM was 0.898.

#### Correlation between OCT Premium (at OCTPre50 and OCTPre3) and MCM performed at Lab A and Lab B for the detection of Trichostrongylidae and coccidia

**Figure 6:**
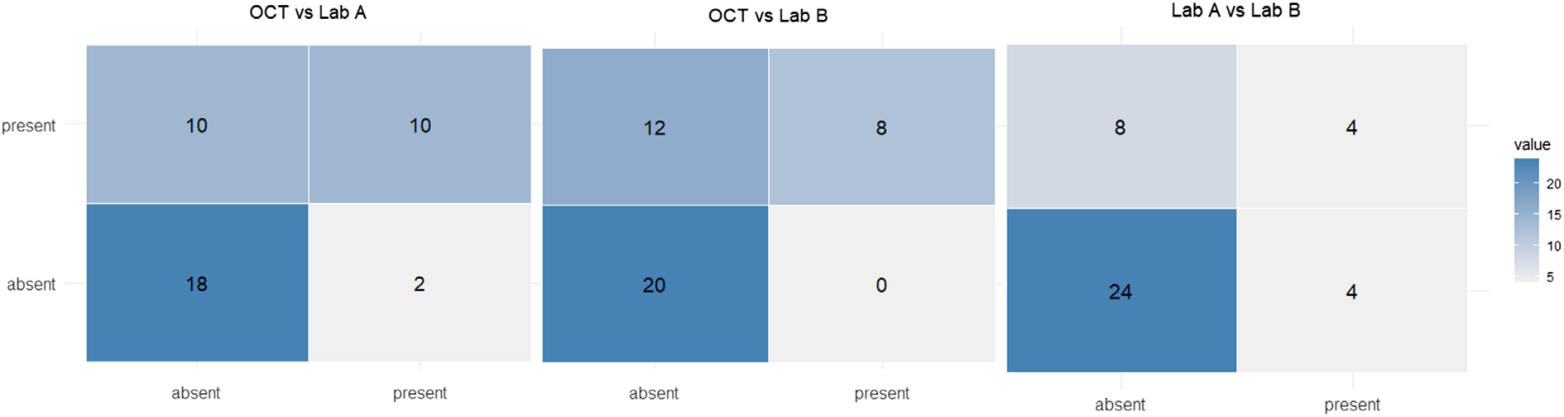
Confusion matrix comparing OCT Premium with the McMaster technique performed in reference laboratories (Lab A: McMaster at Laboratory A; Lab B: McMaster at Laboratory B), based on the presence/absence classification of coccidia type B.

The correlation between different techniques for the detection of Trichostrongylidae and coccidia is presented in Table 4 and Figure 6. For Trichostrongylidae EPG, the OCTPre3 showed the strongest correlation with MCM Lab B (*r*_*s*_ = 0.94) followed by MCM Lab A (*r*_*s*_ = 0.89) with statistical significance (*p* < 0.001) for both correlations. When the cut-off was increased to 50 EPG, the correlation decreased slightly; however, it remained very strong between OCTPre50 and MCM Lab B (*rₛ* = 0.88) and between OCTPre50 and MCM Lab A (*rₛ* = 0.78). The correlation between MCM Lab B and MCM Lab A was also very strong (*rₛ* = 0.84).

**Table 4:** Spearman correlation with 95% confidence intervals (CI%) between OCT Premium, MCM Lab A (McMaster performed at laboratory A), and MCM Lab B (McMaster performed at laboratory B) for detecting Trichostrongylidae at a cut-off above 3 egg per gram (EPG) and 50 egg per gram (EPG) and for coccidia oocyst per gram (OPG).

Techniques	Parasites		
	Trichostrongylidae (cut-off above 3 EPG)	Trichostrongylidae (cut-off above 50 EPG)	Coccidia OPG
OCT Premium & Lab B	0.94 (0.88–0.97)	0.88 (0.75–0.94)	0.77 (0.59–0.87)
OCT Premium & Lab A	0.89 (0.79–0.94)	0.78 (0.56–0.89)	0.65 (0.41–0.80)
MCM Lab B & MCM Lab A	0.86 (0.74–0.93)	0.69 (0.41–0.85)	0.39 (0.08–0.63)

The correlation for coccidia OPG indicated a strong positive correlation between OCT Premium and MCM Lab B (*r*_*s*_ = 0.77). Additionally, strong positive correlations were observed between OCT Premium and MCM Lab A (*r*_*s*_ = 0.65). In contrast, the correlation between MCM Lab A and MCM Lab B was the weakest (*r*_*s*_ = 0.39).

#### Correlation and percentage agreement between OCT Premium, MCM Lab A, and MCM Lab B for the presence of coccidia type B

The correlation results for the presence of coccidia type B, based on OCT Premium and MCM from both Lab A and Lab B, indicate moderate correlation *(rₛ* = 0.50). In contrast, weak correlation was observed between MCM Lab A and MCM Lab B, with *rₛ* = 0.15. The Cohen’s Kappa agreement between OCT Premium and MCM Lab A yielded a Kappa value of 0.40 (95% CI: 0.17–0.63). Similarly, the agreement between OCT Premium and MCM Lab B also showed a Kappa of 0.40 (95% CI: 0.17–0.63). In contrast, the agreement between MCM Lab A and MCM Lab B was lower, with a Kappa of 0.21 (95% CI: –0.11–0.53).

The percentage agreement between different techniques for detecting coccidia type B (at cut-off 100 OPG) varies, as shown in Figure 6. The results indicate that 55% of cases have agreement across all three techniques. The agreement was higher, with 70% concordance observed between OCT Premium and MCM Lab A, OCT Premium and MCM Lab B, and MCM Lab A and Lab B. Among the 12 positive cases identified by MCM Lab A, two were only picked up by this laboratory and were not confirmed by the other techniques. In contrast, all eight positive cases detected by MCM Lab B were also confirmed by OCT Premium. Overall, OCT Premium estimated the highest number of positive cases, identifying 20 out of 40, compared with 12 out of 40 for MCM Lab A and 8 out of 40 Lab B, Figure 6.

### Analytical performance

#### Analytical performance of OCT^+^ in comparison to MCM and MF for detecting gastrointestinal parasites

A summary of the precision of the three different diagnostic techniques (OCT^+^, MCM, and MF) in detecting various parasite eggs in the mixed ovine sample is represented in Table 5. OCT^+^ demonstrated the highest precision for coccidia (92%), strongyles (94%), and *S. papillosus* (75%). MCM showed 89% precision for coccidia, 85% for strongyles, and 33% for *S. papillosus.* MF achieved 88% precision for strongyles, 70% for *S. papillosus*, and 77% for coccidia. The LOQ and LOD values differed across the techniques, with OCT^+^ generally yielding lower values than MF and MCM, especially for coccidia, strongyles, and *S. papillosus*.

**Table 5:** Precision (*P*%), *R* squared value (*R*^2^), limit of detection (LOD), and limit of quantification (LOQ) results for various ovine parasites using OCT Plus (OCT^**+**^), McMaster (MCM), and Mini-FLOTAC (MF).

Parasite	OCT^+^				MCM				MF			
	*P*%	*R* ^2^	LOQ	LOD	*P*%	*R* ^2^	LOQ	LOD	*P*%	*R* ^2^	LOQ	LOD
Coccidia	92	1.00	0.032	0.011	89	1.00	0.156	0.051	77	1.00	0.053	0.017
Strongyles	94	1.00	0.062	0.021	85	1.00	0.158	0.052	88	1.00	0.069	0.023
*Moniezia* spp.	–	1.00	0.330	0.109	–	0.00	0.330	0.109	–	0.00	0.330	0.109
*Nematodirus* spp.	–	1.00	0.124	0.041	–	0.50	0.330	0.109	–	1.00	0.194	0.064
*Strongyloides papillosus*	75	0.98	0.091	0.030	33	0.50	0.330	0.109	70	1.00	0.330	0.109

#### Analytical performance of OCT Premium in comparison to MCM Lab A for the presence of both for the detection of Trichostrongylidae and coccidia

The precision results showed that OCT Premium had higher precision compared to MCM Lab A for both Trichostrongylidae and coccidia parasites, as shown in Table 6. In the same sample repeated 10 times, OCT Premium demonstrated 79% and 82% precision for Trichostrongylidae and coccidia, respectively compared to 48% and 66% for MCM Lab A.

**Table 6:** Precision comparison including mean, standard deviation (±SD) of faecal egg counts, and precision (*P*%) for detecting Trichostrongylidae and coccidia between OvaCyte Premium (OCT Premium) and McMaster performed at laboratory A (MCM Lab A).

Parasite	OCT Premium			MCM Lab A		
	Mean	±SD	*P*%	Mean	±SD	*P*%
Trichostrongylidae	636.5	±130.71	79	545	±281.31	48
Coccidia (*Eimeria* spp.) OPG	352.4	±62.39	82	445	±151.74	66

## Discussion

This study provided insight into the current performance of the OCT compared to commonly used quantitative diagnostic reference techniques. The variability between these manual reference techniques carried out in-house, MCM and MF, were minimised by the fact that they were conducted by the same expert parasitologist in a laboratory under optimal conditions. To avoid variation in quantitative results between techniques due to differences in sample preparation or homogenisation, a single prepared solution was used in our internal trials comparing MCM, MF, and OCT^**+**^. This approach was to ensure a true comparison, allowing for accurate validation of egg counts across all three techniques. Overall, OCT^**+**^ demonstrated higher Se than the other two methods for most parasites, indicating that it is a useful tool for detecting infections. MCM consistently showed lower Se, particularly for strongyles, *Nematodirus* spp., and *S. papillosus*, which could lead to false negatives. MF performed better than MCM, but was generally less sensitive than OCT^**+**^. This can be explained by the lower detection thresholds of OCT^+^ (3 EPG) compared to MF (7.5 EPG) and MCM (50 EPG). Our findings are consistent with previous evaluations of the original OCT version. For instance, an early bovine study in 2020 [14] reported that OCT was equivalent to both MCM and MF in detecting and quantifying strongyle eggs. More recently, Elghryani *et al.* (2023) [15] demonstrated that OCT exhibited Se and Sp that were statistically comparable to those of both MCM and MF. Research has demonstrated that certain traditional techniques, such as the MF technique, can provide higher sensitivity compared to other conventional approaches, likely due to their lower detection thresholds. For instance, a study assessing helminth egg examination in camel faecal samples found that the MF detected higher frequencies of strongyle eggs (68.6%) compared to the MCM (48.8%) and semi-quantitative flotation techniques (52.7%) [24]. Other studies highlight the fact that MF is shown to be more accurate than MCM [2, 12].

Accurate FEC estimation is essential, especially when conducting the faecal egg count reduction test (FECRT), which is a technique recommended by the World Association for the Advancement of Veterinary Parasitology (W.A.A.V.P.) for detecting anthelmintic resistance at the farm level [20]. To enhance diagnostic sensitivity, using a technique with the lowest multiplication factor is recommended, enabling precise identification of animals with zero EPG, accurate assessment of drug efficacy, and improved individual selection for targeted treatment strategies [20].

In this study, OCT^**+**^ detected a higher percentage of strongyle-positive cases than the other two techniques, identifying 70% positivity, compared to 63.5% with MF and 47.6% with MCM. This is illustrated in Figure 2, where the comparison between MF, MCM, and OCT^**+**^ revealed that differences in coccidia and strongyles count estimates became increasingly pronounced as parasite counts increase. Under conditions of low coccidia levels (0–100 OPG), both OCT and MCM closely aligned with MF, indicating strong agreement among the methods. However, for strongyle counts, the divergence increased with parasite burden, as shown in Figure 2A. While differences are minimal in the 0–100 range, OCT^+^ displays higher median counts than MF in the 501–1000 and 1001–2000 ranges, suggesting enhanced sensitivity. In contrast, MCM reports lower counts. A similar trend was observed for coccidia counts, where OCT^+^ also demonstrated higher positive deviations, particularly in the 1001–2000 range, as shown in Figure 2B. In contrast, MCM again displayed progressively larger negative median differences as the count range increased. This aligns with the LOD and LOQ results (Table 2), where OCT^**+**^ proved to be the most effective technique, exhibiting the highest precision, ranging from 75% to 94% and an LOD of < 0.03 across most parasites [coccidia (0.01), strongyles (0.02), and *Strongyloides* spp. (0.03)]. In contrast, MCM showed the weakest performance, particularly for *S. papillosus* (33%) and *Nematodirus* spp. (0.50%), making it less reliable for diagnosing these parasites, especially since in most samples, very few eggs were detected. The high precision and lower LOD values of OCT^**+**^ demonstrate its improved ability to detect parasites, especially in low egg/oocyst counts. These findings are comparable with previous studies [1, 21, 36, 37], which highlight that traditional flotation techniques, such as MCM, are less reliable for detecting parasite eggs/oocysts when present in low numbers.

The correlation analysis revealed strong positive relationships between OCT Premium and the MCM results from Lab A and Lab B, particularly for Trichostrongylidae EPG and coccidia OPG, where the counts were generally higher and exceeded the MCM method’s threshold of 50 EPG, highlighting the reliability of OCT Premium in FEC. The strong correlations indicate that OCT Premium could be a robust and reliable alternative to manual MCM, providing high precision with minimal variability. The precision results showed that OCT Premium exhibited more consistent performance than MCM results of Lab A in detecting both coccidia and Trichostrongylidae parasites. Across ten repeated measurements on the same sample, OCT Premium demonstrated higher precision, achieving 82% for coccidia and 79% for Trichostrongylidae, whereas MCM results of Lab A showed significantly lower precision, with 66% and 48%, respectively. The higher precision of OCT Premium indicates greater measurement stability, minimising the influence of random errors or inconsistencies commonly observed in traditional techniques such as the MCM. The inconsistencies in MCM results can be attributed to human error and variability in reading individual MCM slides, influenced by factors such as extent of formal training, time constraints, rushed slide movements, and fluctuation in mental focus [4, 36]. In contrast, OCT^**+**^ and OCT Premium applies the same automated intelligence approach across all samples. Another factor contributing to the high precision of OCT^**+**^ and OCT Premium is the unique concentration properties of the OCT cassette, that enhance the volume detected, which is greater than the MF and MCM chamber volumes. It has been reported in various studies that analysing a larger suspension volume provides a more representative assessment of the faecal sample, which, in turn, is expected to improve precision [3, 4].

Recent advances in AI have significantly contributed to the development of automated diagnostic tools for parasite egg identification and quantification. Several AI-powered systems employing imaging techniques and computational analysis have highlighted higher diagnostic accuracy compared to traditional techniques [6, 8, 18]. Bucki *et al.*, 2023 [3] conducted a study on ovine faecal strongyle egg counts, comparing the Micron kit to MCM and finding that the Micron kit yielded significantly higher EPG values. Scare *et al.*, 2017 [34] investigated a smartphone-based automated technique to generate automated egg counts, employing imaging procedures and analytical methods to identify and quantify strongyle eggs in horse faecal samples. The study demonstrated that the smartphone system exhibited comparable accuracy to the MCM technique and was more precise than the MF and MCM techniques (*p* < 0.0001). Additionally, MF showed greater precision than MCM (*p* = 0.0228). Similarly, Cringoli *et al.*, 2021 [11] evaluated the Kubic FLOTAC microscope, using 30 cattle faecal samples experimentally infected with GI nematodes, showing strong agreement (concordance correlation coefficient = 0.999) with a traditional MF.

AI-driven tools have transformed parasitology by enhancing the diagnostic performance of FECs, particularly in terms of precision, efficiency, and reliability. Using machine learning algorithms and computer vision technology, this system can analyse samples with higher precision, reducing variability caused by subjective human interpretation [6, 40]. Additionally, where there are high egg/oocyst counts, AI systems can process large sample volumes in a shorter time, significantly improving diagnostic efficiency.

The increasing integration of computational technologies in diagnostic parasitology underscores the need for extensive visual and textual databases to support AI model training and validation. AI-based classification systems utilise supervised learning techniques, including Bayesian classifiers [7] and Artificial Neural Networks (ANNs) [25, 41] to improve automated parasite identification. Research such as Yang *et al.*, 2001 [41] developed an algorithm that uses digital image processing and an ANN classifier to analyse faecal samples containing seven species of human helminth eggs. The key morphometric parameters in this study represented shape, shell smoothness, and size. The algorithm achieved a detection rate of 84% based on the classification of 82 images. While Castañón *et al.* [7] developed an automated feature extraction technique to analyse the shape of seven *Eimeria* species in domestic fowl, focusing on curvature, geometry, and texture. A 13-dimensional feature vector was created for each oocyst image, and species identification was performed using a Bayesian classifier. The method achieved 85.75% accuracy using a dataset of 3,891 micrographs for training.

A study by Singh *et al.* [35] highlighted the potential of deep learning-based mobile applications for automated identification of *Eimeria* and *Cystoisospora* species in pigs. Using convolutional neural networks, specifically resource-efficient models like MobileNetV2 and EfficientNetB1, the study achieved high segmentation and classification accuracy, with MobileNetV2 reaching an m-IoU of 0.95 and 93% classification accuracy.

As AI continues to evolve, its integration into parasitology is expected to enhance disease monitoring, improve treatment strategies, and contribute to more effective parasite control programmes. Given the importance of rapid and accurate diagnostics in parasite prevention, this study validates automated OCT Premium for detecting *H. contortus* eggs, demonstrating higher sensitivity than manual MCM results from the accredited Lab A, with the PNA technique used as the true reference. Our findings with OCT Premium aligned with PNA results where 31 out of 40 samples were contaminated by *H. contortus*. Recent studies have investigated automated techniques for detecting and quantifying *H. contortus* using PNA-stained eggs and a multimode reader, which allow for faster and more accurate diagnosis [13]. Castle *et al.* [8] demonstrated that the Parasight System, adapted for *H. contortus* detection with PNA staining, showed strong correlations with manual PNA, confirming its accuracy and efficiency. However, these systems require egg staining during sample preparation, resulting in a lengthy testing process for each sample. In contrast, OCT Premium offers a more straightforward technique, eliminating the need for specialised laboratory equipment or technical expertise. It enables rapid sample testing without the need for laboratory referral, while its technology enables image transmission, supporting future advances in automated egg identification and counting.

The presence of *N. battus* and coccidia type B (*E. weybridgensis, E. crandallis*, and *E. ovinoidalis*) in faeces can be detected by microscopic identification of eggs/oocysts. Culturing of coccidia oocysts can also be carried out to assist in species-level identification. However, accurate morphological identification of *N. battus* eggs and coccidia type B oocysts in faecal samples requires expertise from trained technical staff. The advanced version of OCT Premium has been further developed to distinguish *N. battus* eggs and coccidia type B oocysts based on distinct morphological characteristics, including colour and size, improving detection accuracy and reducing reliance on manual expertise. This approach aligns with advances in automated diagnostic tools for parasite detection. For instance, Castañón *et al.*, 2007 [7] described the use of computer-assisted image analysis for the detection and quantification of *Eimeria* oocysts in poultry faeces based on statistical evaluation of size and shape. Similarly, Vineer *et al.*, 2025 [33] introduced a machine learning-based platform for automated identification of multiple *Eimeria* species in ruminants. These methods highlight the increasing role of image-based and AI-supported technologies in enhancing diagnostic efficiency and accuracy across different host species.

### Limitations

The study’s limitations includes sample size and restricted sample location, with all samples collected from farms in Ireland.

## Conclusions

The OCT system presented in this study is well-suited for large-scale laboratory tests, ensuring consistent performance throughout. Of the OCT Premium evaluations in this study, only the binary results for the presence of *H. contortu*s in a sheep sample were validated. Further studies are underway to evaluate the quantitative performance for *H. contortus* in a sample.

## Figures and Tables

**Figure 1:**
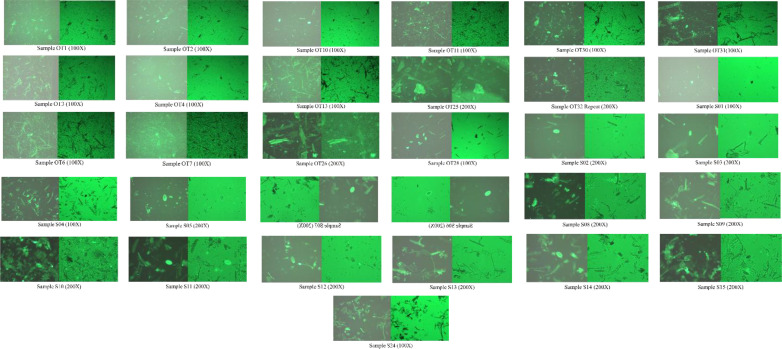
Images of *Haemonchus contortus eggs* under fluorescent microscope from *Haemonchus contortus*-infected sheep samples, with (left) and without (right) a UV filter.

**Figure 2:**
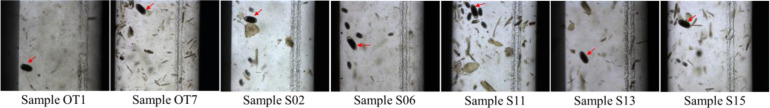
Scan images of *Nematodirus battus* eggs (indicated by red arrow) of OCT Premium from of all the *Nematodirus battus* infected sheep samples.

**Figure 3:**
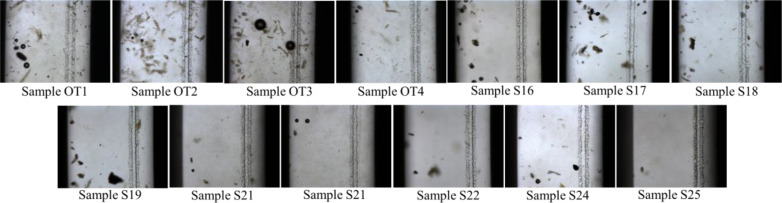
Scan images of Trichostrongylidae eggs of OCT Premium from infected sheep samples.

**Figure 4:**
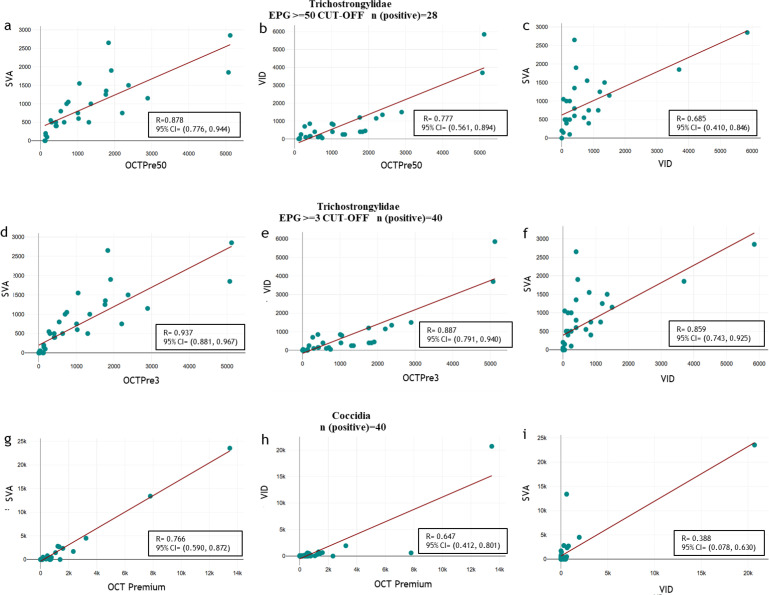
Spearman correlation coefficient (95% CI) for the detection of Trichostrongylidae: (a, b, c) among OCTPre50, Lab A and Lab B; (d, e, f) among OCTPre3, Lab A and Lab B. Spearman correlation coefficient (95% CI) for the detection of coccidia: (g, h, i) among OCT Premium, Lab A and Lab B.

## Supplementary material

The supplementary material of this article is available at https://www.parasite-journal.org/10.1051/parasite/2026002/olm.
